# Alcohol and Periodontal Disease: A Narrative Review

**DOI:** 10.7759/cureus.62270

**Published:** 2024-06-12

**Authors:** Utsav H Gandhi, Amit Benjamin, Shreya Gajjar, Tanvi Hirani, Khushboo Desai, Bansariben B Suhagia, Rahnuma Ahmad, Susmita Sinha, Mainul Haque, Santosh Kumar

**Affiliations:** 1 Department of Periodontology, School of Dentistry, Karnavati University, Gandhinagar, IND; 2 Department of Periodontology, Ahmedabad Dental College and Hospital, Gujarat University, Ahmedabad, IND; 3 Department of Physiology, Medical College for Women and Hospital, Dhaka, BGD; 4 Department of Physiology, Enam Medical College and Hospital, Dhaka, BGD; 5 Department of Research, Karnavati Scientific Research Center, School of Dentistry, Karnavati University, Gandhinagar, IND; 6 Department of Pharmacology and Therapeutics, National Defence University of Malaysia, Kuala Lumpur, MYS

**Keywords:** necrotizing periodontal diseases, alcohol-induced carcinoma, tooth decay, tooth cavity, alveolar or bundle bone, normal gamma-glutamyl transferase (ggt), periodontitis, gum disease, liquor, booze

## Abstract

The scientific literature dealing with alcohol and alcoholic beverages revealed that these drinks possess an adverse impact on periodontal tissues. Additionally, other principal risk factors include tobacco, smoking, poor oral hygiene, etc. It has been observed that among chronic alcoholics, there are further issues, such as mental, social, and physical effects, that promote alcoholism. These people may have weak immunity for defense against pathogenic organisms and bacteria. Thus, chances of gingival bleeding, swollen gums, bad breath, and increased bone loss are there. Different alcoholic beverages in the market cause less salivation; these beverages contain sugars that promote acid production in the oral cavity by pathogens that demineralize the enamel and damage gum and teeth. This chronic alcohol consumption can progress into different types of oral disorders, including cancer, halitosis, and caries, and is also associated with tobacco and smoking. Chronic alcohol consumption can cause alteration of the oral microbiome and increase oral pathogens, which lead to periodontal disease and an environment of inflammation created in the body due to malnutrition, diminished immunity, altered liver condition, brain damage, and gut microbiota alteration. Heavily colored alcoholic beverages produce staining on teeth and, due to less saliva, may cause other toxic effects on the periodontium. Over-dependency on alcohol leads to necrotizing lesions such as necrotizing gingivitis, necrotizing periodontitis, and necrotizing stomatitis. These pathological impairments instigate severe damage to oral structures. Therefore, proper counseling by the attending dental surgeon and related health professionals is urgently required for the patient on the basis that the individual case needs to go away from the regular heavy consumption of alcohol.

## Introduction and background

Periodontal diseases include inflammatory conditions that affect the supporting structures of the teeth, including the gingiva and bone, which may lead to tooth loss and other systemic inflammation [1,2]. It has been reported that worldwide, 5-50% of the population suffering from periodontitis eventually lose their teeth [3-5]. Some of the behavioral factors that can cause periodontal diseases include smoking [6-8], chronic alcohol consumption [9,10], nutritional deficiency [11,12], psychological issues [13,14], and systemic diseases [15-17].

In this narrative review, the authors are going to discuss the effects of alcohol consumption on oral tissues that lead to periodontal diseases. A regular and considerable amount of alcohol consumption is more commonly seen in middle and older age groups compared to younger ones [18,19]. Nonetheless, recently, the scenario started changing in the younger community frequently, with a sizable portion consuming alcoholic drinks globally, which also includes India [20-23]. Alcohol causes more negative effects on older people because of biological changes, especially in the brain [24], conciliation renal physiology [25], pharmacokinetic and pharmacodynamic changes [26], and increased need for medication because of the aging process [27,28]. Alcoholism-induced periodontal disease can be associated with poor oral hygiene [29]. In most cases, poor periodontal health is due to poor oral hygiene or not taking care of dental care rather than alcohol use [4,30,31].

Yuan et al. reported that two healthy régime features comprise refraining from substantial alcohol drinking and persistent eating and drinking practices reformed Mediterranean diet [32]. Multiple studies reported that drinking alcohol, high body mass index (BMI), cigarette smoking, unwholesome diet, and physical sedentariness remain as principal modifiable risk factors of lifestyle that cause multiple diseases [33,34]. For a considerable portion of the population globally, including in low-and-middle-income countries (LMICs), consistently consuming alcohol becomes one of the critical reasons for seriously damaged health [35,36]. These patients often take less interest in dental health care and do not think seriously about any advice dental health professionals provide [37-39].

Problem statement

Chronic alcoholism possesses the potential to harm the general health and well-being of a person [40,41]. Alcohol necessity and habit are categorized by primary transformations in the brain's compensation (incentive) and anxiety (worry) systems that are discernable as withdrawal symptoms when alcohol drinking is ceased or noticeably cut down. These transformations are also ostensible to fire impetus to enroll again in extreme alcohol consumption rate [42,43]. In 2016, it was reported that alcohol abuse was the seventh leading cause of disability and premature mortality [44,45]. Chronic high amounts of alcohol consumption frequently lead to hypertension, cardiac and hepatic disorders, stroke, and gastrointestinal diseases. Additionally, there is a strong association in developing the mouth, breast, esophagus, throat, voice box, liver, colon, and rectal carcinoma [46]. Chronic and heavy alcohol drinking indeed distresses the mouth cavity, oral mucosa, and teeth [47]. Additionally, it has been observed that a substantial portion of alcohol consumption is associated with solid periodontal health. It has been clarified by several biotic probabilities, such as an increased possibility of severe infection (ruins neutrophil, macrophage, and T-cell physiological function) due to compromised immune systems and unhealthy oral hygiene practices [48-52]. Priyanka et al. 2017 reported that the "prevalence of periodontitis was higher (89.61%) in alcohol-dependent subjects compared to controls (78.67%). Prevalence of mucosal lesions among alcohol-dependent subjects was 31.5%, which was higher than the controls (25%)" [29]. The underlying mechanisms for the causation of periodontal diseases in alcoholics and the association of alcoholism with other harmful habits also need further investigation.

Objectives of the study

This paper intends to evaluate and describe the effect of alcoholism on periodontal tissues. Additionally, this study plans to appraise the role of alcoholism in the pathogenesis of periodontal diseases and assess the relationship of alcoholism with other harmful habits, such as smoking, on periodontal tissues. This narrative essay forms an opinion of the role of alcohol-containing mouthwashes in maintaining proper oral hygiene.

## Review

Materials and methods

**Figure 1:**
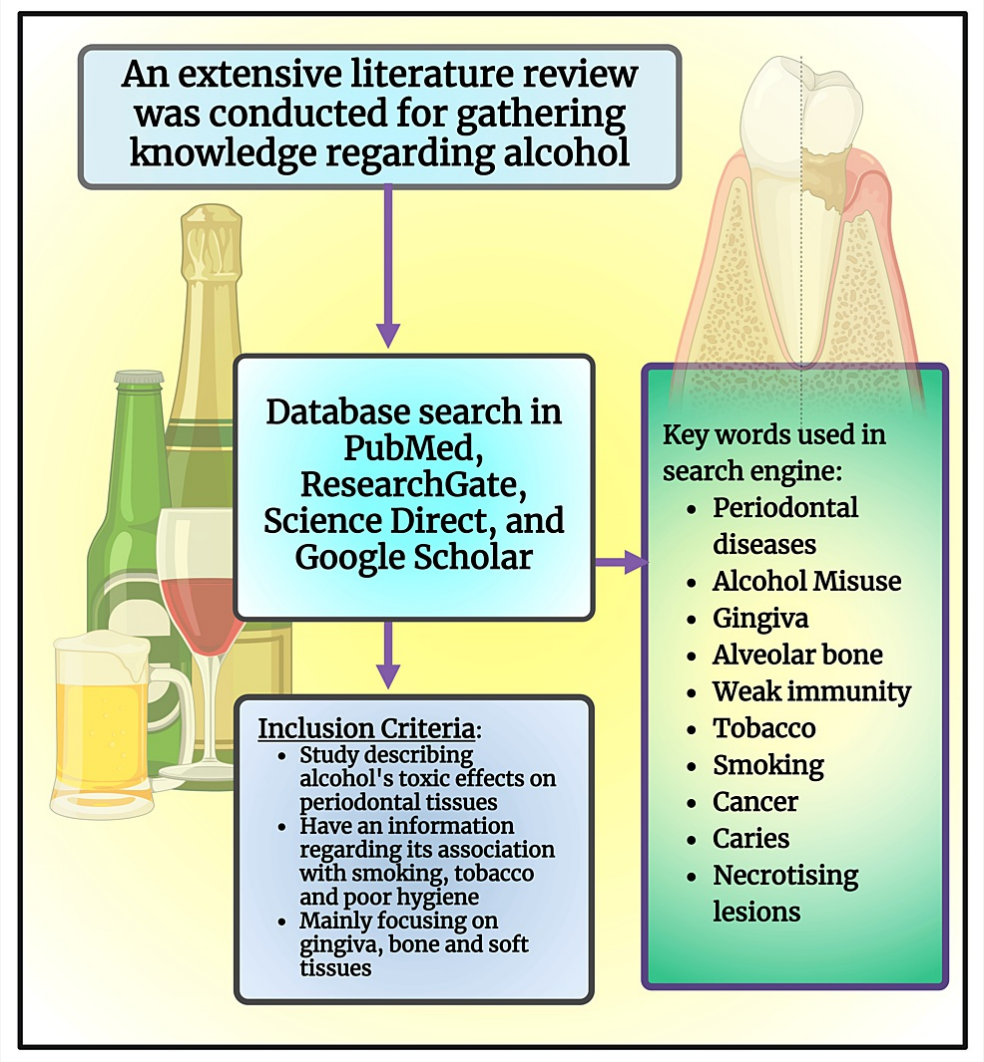
Schematic diagram illustrating the methodology of the study. Note: This figure was drawn using the premium version of BioRender (https://biorender.com/ (Accessed on 25th April 2024)) with the agreement license number VO26QPXSCW [53]. Image credit: Susmita Sinha

This review considered data in a database (PubMed, Google Scholar, Elton B. Stephens Company (EBSCO)), year (2000 to 2023), language (English), and study design (Systematic Reviews, Narrative Reviews, Observational Studies). Researchers utilized keywords such as "Periodontal diseases," AND "Alcohol misuse," AND "Gingiva," AND "Alveolar bone," AND "Weak immunity," AND "Tobacco," "Smoking," AND "Cancer," AND "Caries," AND "Necrotizing Lesions." Inclusion criteria are studies describing alcohol's toxic effects on periodontal tissues, association with smoking, tobacco, and poor oral hygiene, mainly focusing on the gingiva, bone, and soft tissues (Figure 1). Exclusion criteria are studies in other languages, animal and in vitro studies, and studies involving other medically compromised patients.

Discussion

Gamma-glutamyl transferase (GGT) is a liver enzyme. Nevertheless, this enzyme is integrated into "the plasma membranes of most cells and organ tissues" [52]. GGT levels are substantially raised among individuals drinking alcohol in excessive quantity and persistently [54,55] and denote the severity of periodontal disease [56,57]. In some studies, the use of alcohol is associated with self-reported periodontal disease, especially among tobacco smokers [58-60]. A robust relationship was detected among tobacco smokers in males and females who drink alcohol more than 30 g and 20 g daily, respectively [58]. Individuals who meet a major portion of total dietary energy intake through excessive alcohol consumption are often found to suffer from primary and secondary malnutrition [61-63]. In primary malnutrition, alcohol supplants or substitutes some nutritional intake - triggering a paucity of essential elements, vitamins, minerals, and proteins [61,63,64]. In secondary malnutrition, alcohol leads to gastrointestinal problems and causes malabsorption and malnutrition [65].

Consistent alcohol intake increases the possibility of infections [66]. High levels of alcohol drinking for a prolonged time often lead to protein deficiency [67,68]. Complement instigation is the principal issue of the innate immune system, which plays an imperative function in protecting against microbial infectious diseases, restoring the host, and clearing the inflammatory damage products [69,70]. Complement insufficiencies are caused by the specific complement protein deficiency [71]. Chronic heavy alcohol is frequently found with C3 and C4 protein deficiency [72,73]. Persistent heavy alcohol consumers repeatedly found defective neutrophil function, macrophage, and T-cell function [74]. Thus, chronic alcoholics are highly prone to communicable diseases (bacterial and viral) that include periodontal infections [75-77]. Individuals consuming a high quantity of alcohol relentlessly promote dysbiosis of the oral microbiome [78] and encourage periodontal pathogenic microbes' accumulation [79] and tooth pocket formation more than non-alcoholics [10]. *Porphyromonas gingivalis* and *Fusobacteria nucleatum* are the most notorious periodontal pathogenic microbes [80]. It has been reported that the accumulation of these pathogens (*P. gingivalis* and *F. nucleatum*) was substantially higher among heavy alcoholic drinkers than in non or occasional alcoholics [28,81,82]. Chronic heavy alcohol consumers have a higher overall quantity of red (i.e., *P. gingivalis*) and orange-complex (i.e., *F. nucleatum*) pathogens than non-alcoholic subjects [83]. *P. gingivalis* and *F. nucleatum* repeatedly cause severe periodontitis with tooth loss [84,85].

Effects of alcohol on alveolar bone

A J-curve has been anticipated regarding the implication between alcohol consumption and springing up and the evolution of periodontal infectious diseases. It was explained that consuming alcohol in small quantities is prone to minor evolution and high doses with a more hostile and destructive advancement of the disease. Therefore, adverse outcomes are dose-dependent [86-89]. Long-standing drinking of alcohol blights the equilibrium of microbiota in the digestive tract, causing the gastrointestinal physiological impediment [78,90,91], the liver's capability to decontaminate microbial yields and to spawn a well-adjusted cytokine ambiance, and the brain's authority to control inflammation in the periphery. While these protection lines are compromised, systemic inflammation proceeds [90].

**Figure 2:**
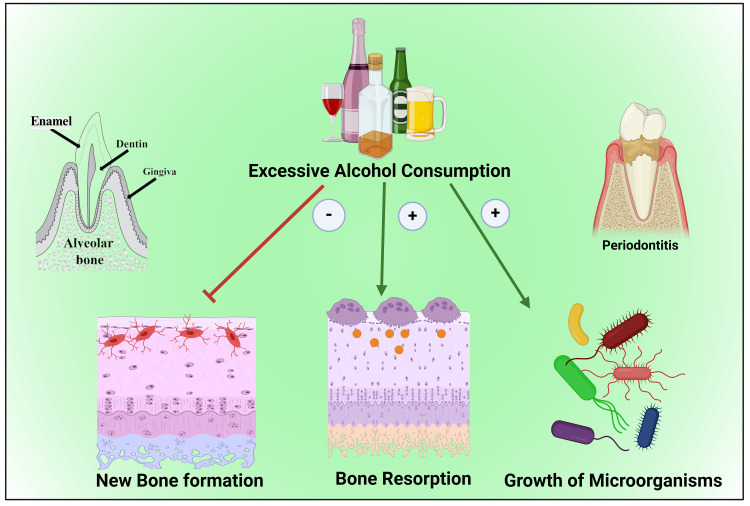
Illustration showing the detrimental effect of alcohol consumption on alveolar bone. Note: This figure was drawn using the premium version of BioRender (https://biorender.com/ (Accessed on 19th May 2024)), which has the agreement license number CR26U3IFYP [53]. Image credit: Susmita Sinha

Moreover, the sugar content of alcohol attracts pathogenic microbes to accumulate in the oral cavity [79,92,93]. The prolonged high amount of alcohol drinking raises the severity of periodontitis in a dose-dependent mode [88,89] (Figure 2) by raising the scale of the inflammatory reaction in a confined area [48]. Those include receptor activator of nuclear factor kappa-Β ligand (RANKL) immunoreactivity outline and the number of tartrate-resistant acid phosphatase (TRAP) positive cells (TRAP+cells), and tumor necrosis factor-α (TNF-α), which provide a route to more prominent alveolar bone knocking down and a lower pulpal blood flow (PBF) [48,94,95].

Alcohol on periodontal tissues

Heavy regular alcohol drinking directly affects the bone anabolism [12,95]. Alcohol instigates the clampdown of osseous tissue construction because of its toxic consequence on osteoblastic endeavor and multiplying [96,97]. Alcohol inhibits osteoblastic pursuit but promotes osteoclastic accomplishment [97-99]. Additionally, alcohol affects the gastrointestinal system, causing malnutrition [63,65]; thereby, multiple essential nutritional components for the growth and proliferation of bone are not absorbed [61]. Moreover, heavy alcoholic beverage consumers often meet their energy needs up to 60% [100]. Therefore, essential nutritional intake is usually deficient, which affects bone growth [100,101].

Alcohol and the development of dental caries

Alcohol has been reported to trigger many oral health disorders. These include periodontal disorder, tooth deterioration, caries, oral malignancy, halitosis, damage, and tarnishing [102]. It has been reported that Chinese colorless liquor named Baijiu promotes dental caries by distressing the oral microbial ecosystem, exclusively by forming dental pathogens biofilms, because Baijiu contains excessive alcohol [103]. Almost all alcoholic beverages or drinks contain sugar [104]; nevertheless, some possess relatively high amounts (five teaspoons of sugar in 700 mL pack) [105], e.g., sherries, fortified wines, cider, and pre-mixed drinks like alcopops and liqueurs [105]. World Health Organization (WHO) reported that "free sugars are the essential dietary factor in developing dental caries" [106]. Sheiham and James reported a solid log-linear association between dental caries and sugar consumption among individuals who consume 0-10% of their energy intake with sugar [107]. Pathogenic microbes within the oral cavity and supragingival plaque utilize sugar as energy through fermentation, producing acid through the Embden-Meyerhof-Parnas (EMP) pathway. This acid demineralized teeth enamel dentin and formed cavities [108-111]. It is not only sugar-containing alcoholics that cause dental carries formation; nonetheless, all sugar-sweetened beverages (SSBs) cause a similar phenomenon [112,113]. WHO reported that fluoride consumption has equally advantageous properties - in dropping the frequency of dental caries - and adverse consequences - in instigating tooth enamel and skeletal fluorosis after long-standing high subjection [114]. Certain alcoholic drinks frequently contain high levels of fluoride [115,116], which increases the risk of caries.

**Figure 3:**
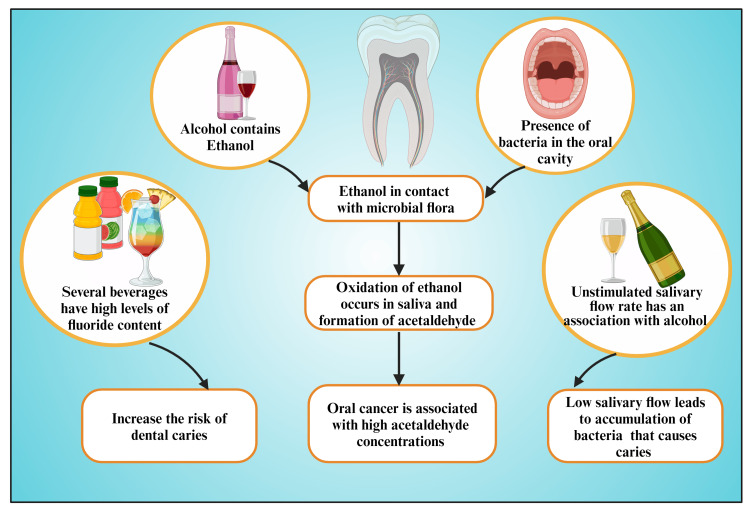
Diagram showing the possible mechanisms by which alcohol consumption results in dental caries. Note: This figure was drawn using the premium version of BioRender (https://biorender.com/ (Accessed on 25th April 2024)), which has the agreement license number HU26QQ80TS [53]. Image credit: Susmita Sinha

A considerable portion of acute and long-standing alcohol consumption severely impacts oral physiological executions [117]. Alcohol in substantial amounts acts as a diuretic [118,119], causing increased urination with the loss of all essential cellular features and succeeding dehydration [118-120]. Hence, alcohol intake escalates thirstiness and causes xerostomia (dry mouth) [117]. Alcohol-induced thirst perception and oral aridness are primarily initiated by the instigation of impairment in the midline of the brain (dedicated to neuroepithelial configuration) around the third and fourth ventricles [117]. This brain area communicates directly with blood and cerebrospinal fluid (circumventricular organs) [117]. Therefore, through that area, polypeptide hypothalamic hormones depart the brain, exclusive of interrupting the blood-brain barrier (BBB) and hypothalamus with a diminished rate of salivary excretion [117]. This lower salivation flow changes pH toward the acidic side [121,122] than normal individuals. Additionally, low salivary flow leads to more microbial accumulation on enamel surfaces [123] and synthesizing more acid that causes caries [124] (Figure 3).

Alcohol-instigated carcinoma

Alcohol-instigated cancer incidence rates were among males (3%) and females (0.5%), and death rates were 2.8% (male) and 0.1% (female) in Korea, respectively [125]. The principal cause of mortality among people of the Republic of Korea is cancer, accounting for 26.5% of all fatal outcomes. These cancers are robustly associated with lifestyle issues, e.g., chronic heavy alcohol consumption, high-level tobacco smoking, high BMI level, and low physical activity. This study also revealed that poor style issues increase the possibility of cancer by 1.47 times more threat than those who maintain a healthy lifestyle [126]. A substantial proportion of cancer is frequently ascribable to alcohol consumption, especially among regular and heavy in-takers who often cross the upper limits of the recommended dose in Western Europe. Alcohol-caused cancer in European males and females was detected with incidence rates of 10% and 3%, respectively. These cases were erstwhile and existing heavy and alcohol consumers [127]. There is an increased risk (15%) of developing oral, pharyngeal [128,129], esophageal, and colorectum cancer [130] among people who drink alcohol more than 10 g/day [128]. Bagnardi et al., in their meta-analysis, reported that the relative risk of oral and pharyngeal carcinoma was swelled up from 1.13 to 5.13 for existing alcohol consumption around 12.5 g and above 50 g alcohol daily, respectively [131]. Population attributable fraction (PAF) refers to the proportion of all cases with a particular outcome in a population that could be prevented by eliminating a specific exposure [132]. The PAF due to alcohol is 29.3% for oral cavity cancer, 43.3% for pharyngeal cancer, and 25.8% for laryngeal cancer in men. In women, PAF is 4.2% for colorectal cancer and 0.2% for breast cancer [125]. There is three times more permanent teeth loss among alcoholics than among the healthy population [29]. Thus, alcoholics have a lesser number of teeth [133].

Association between tobacco and alcohol

Smoking is the second principal hazardous aspect for mortality and disability-adjusted life-years (DALYs), which has elicited over 200 million people expiries prematurely over 30 years [134,135]. Tobacco chewing is considered independently as a risk factor for oral cancer [136,137]. Tobacco smokers or chewers, along with betel nut and leaf, who concurrently drink alcoholic beverages have a greater probability of oral and esophageal carcinoma [138], as well as head and neck cancer (HNC), especially in Southeast Asia [139,140], and become snowballing public health apprehension [141]. Another study revealed that alcohol and tobacco have give-and-take impacts on despot hankering, "subjective responses to fixed-dose alcohol or nicotine administration and self-administration" [142]. Smoking and alcohol ingestion have been stated as considerable risk features for cardiovascular diseases (CVDs), cancers, type 2 diabetes mellitus (T2DM), obesity, bronchial asthma, stress, stroke, and other lifestyle-related noncommunicable diseases, and increasing mortalities [143-145]. WHO reported annually that around the globe, 7 and 3 million people died because of tobacco and alcohol consumption, respectively [146,147]. McCambridge and Morris revealed that alcohol assassinates smaller people around the globe than tobacco. It has been estimated that roughly 5% of total mortalities are attributed to alcohol (ethanol) compared to 8% for cigarettes and other tobacco products) [148].

Alcohol affecting the brain

Long-lasting, heavy alcohol drinking consequences in anatomical alterations that lead to potentially permanent brain impairment with its neurotoxic possessions [149-152]. Straightforwardly, alcohol causes neuro-inflammation and neuronal damage of brain tissue [153,154]. Additionally, alcohol produces an adverse impact on the gastrointestinal microbiome colonies, triggering hepatic encephalopathy [154-156]. The alcohol-persuaded brain damage led to alcohol-induced persisting amnestic syndrome (Wernicke-Korsakoff syndrome (WKS)) and alcohol-related dementia (ARD), which manifest as forgetfulness, intense perplexity, and visual disturbance [151,157-160]. Wernicke encephalopathy (WE) is an acute neuropsychiatric brain ailment instigated by a thiamine absence or deprivation of vitamin B1 [151].

Alcohol impedes the brain's signaling route and upsets the brain's anatomy and physiology [161,162]. Alcohol affects brain cells and converts brain tissues firmer and smaller, especially those areas responsible for "controlling balance, memory, speech, and judgment to do their jobs," raising a greater probability of physical damage and other adverse consequences [161]. Over time, a substantial portion of drinking alcohol has been reported to cause the wasting of brain tissue, fall of brain mass, impairment of neurons, and compromised physiology function of the white matter fiber [161,162]. Similar findings were also reported when drinking alcohol in mild to moderate portions [163-167]. Alcohol has a negative impact on the hippocampus area of the brain by exerting an adverse impact on neural stem cells and adult neurogenesis [168], causing diminished cognitive function. Thereby, among chronic alcoholics, the transfer of memoirs from short-term to long-term storage is impaired. These patients often are unable to recall many events when they are heavily drunk [169-172]. The memory-transferring phenomenon is known as memory consolidation [173]. Long-term drinking of alcohol often led to modifications in moods, feelings (autocorrelation (emotional inertia)), and individuality, in addition to compromised awareness, discernment, acquiring knowledge, studying, and memorial function [162,174].

Alcohol causing more stains on teeth

**Figure 4:**
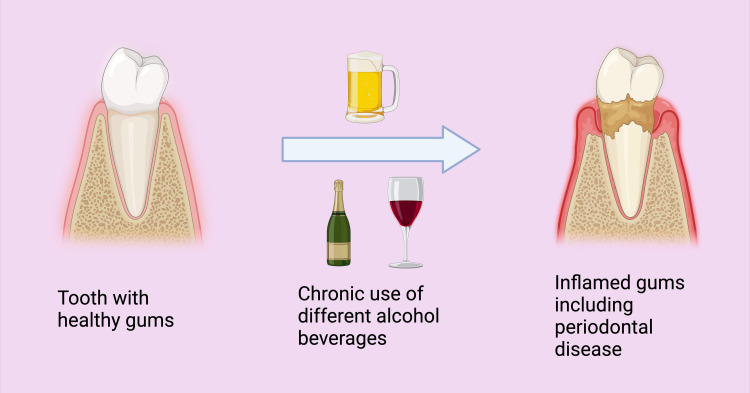
Comparison of healthy teeth with diseased teeth because of misuse of alcoholic beverages. Note: This figure was drawn using the premium version of BioRender (https://BioRender.com/ (Accessed on 23rd April 2024)), which has the agreement license number BQ26QFMZQZ [53]. Image credit: Utsav Gandhi

Certain alcoholic beverages heavily contain coloring agents. Sunset Yellow FCF (Tartrazine, Orange Yellow S, or FD+C Yellow No. 6) is an orange-yellow azo dye usually added to alcoholic beverages. Nevertheless, it also added many SSBs, fat-based desserts, candies, fruit-flavored snacks, drink mixes/powders, and many more foods [175,176]. Sunset Yellow FCF is also used in many pharmaceutical products, such as paracetamol and ibuprofen, which cause tooth discoloration among the pediatric population [177]. Several alcohol-containing cocktails comprise chromogens that produce attractive colors of these alcoholic drinks. These chromogens fasten to dental enamel and discolored teeth habitual consumers (Figure 4) [178].

Necrotizing periodontal diseases

**Figure 5:**
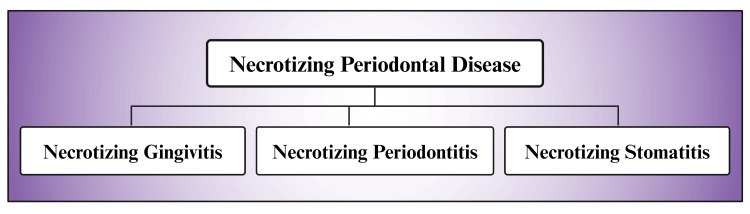
Diagram showing the types of necrotizing periodontal diseases. Note: This figure was drawn using the premium version of BioRender (https://biorender.com/ (Accessed on 25th April 2024)), which has the agreement license number NQ26QQA71P [53]. Image credit: Susmita Sinha

Over-dependency on alcohol, along with its toxic effects, undernourished and poor level of immunity, leads to necrotizing periodontal diseases (NPDs) [9,15,179,180]. Other considerable, affecting aspects causing NPDs include HIV infection leading to compromised immune function, bad oral cleanliness, extraordinary emotional stress, deficient sleep, being Caucasian, age range (18-21 years), tobacco use, vitamin C deficiency, chronic considerable portion alcohol drinking, and recent severe diseases [9, 181,182]. Another study reported that NPDs are interrelated to host immunity status [183]. NPDs are of three types (Figure 5) [180]. NPDs are a group of chronic noncommunicable microbial dysbiotic inflammatory oral/dental illnesses. NPD is portrayed by a speedy commencement of inflammation, soreness, ache, gum hemorrhage, interdental or papilla necrosis, and licked or pounded crater-like contusion of the papilla interdentalis [9,184-190]. NPDs differ from other chronic gingivitis through the presence of considerable pain, gingival necrosis amid the teeth, cankerous papilla, and gum bleeding [191]. The precise pathological process of NPDs yet remains elusive [180].

Alcohol-containing mouthwashes

**Figure 6:**
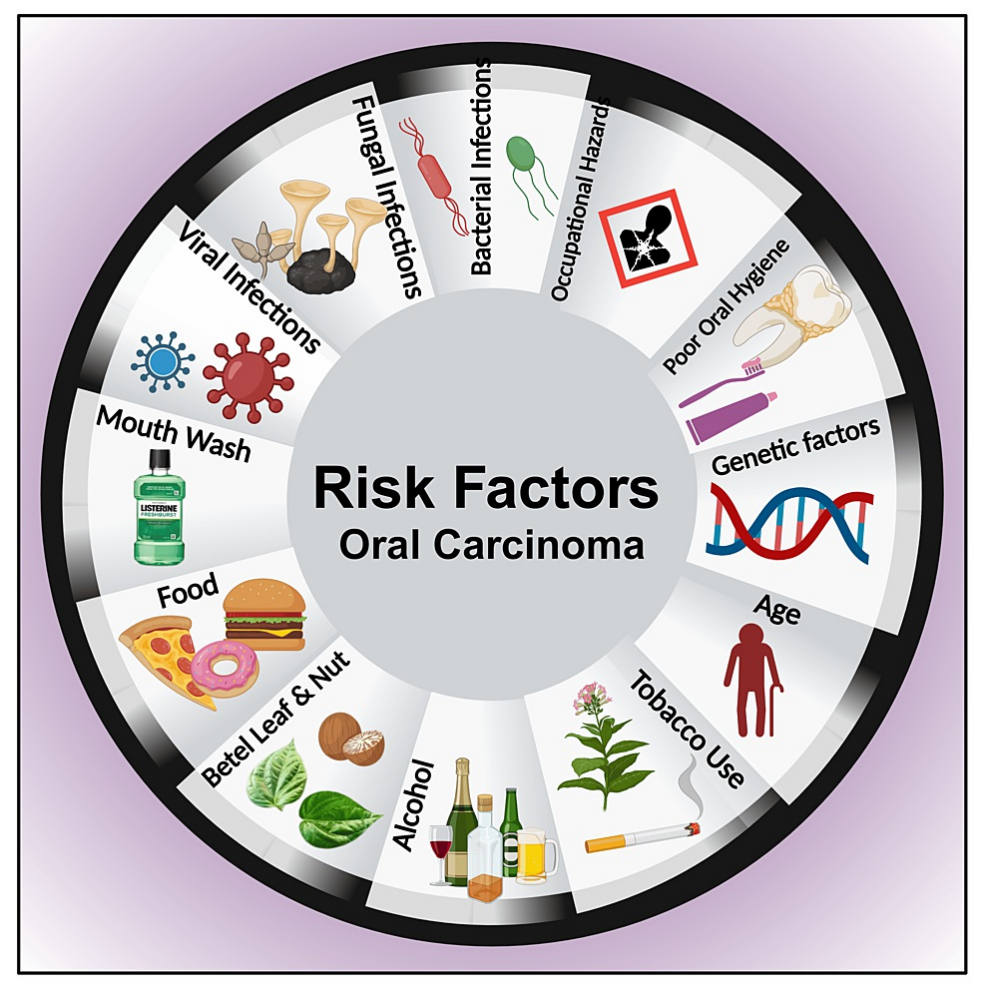
Schematic diagram showing the risk factors for oral carcinoma. Note: This figure was drawn using the premium version of BioRender (https://biorender.com/ (Accessed on 17th May 2024)), which has the agreement license number EQ26TU4XE2 [53]. Image credit: Susmita Sinha

Several brand mouthwashes contain alcohol. It has been reported that ethanol concertation perchance escalated to 26% [192]. Multiple studies earlier reported that containing ethanol mouthwashes increases the possibility of developing oral cancer [193,194]. However, it has been reported that alcohol alone does not comply as an independent risk factor for evolving oral carcinoma; nonetheless, the probability does intensify with the presence of other risk features of mouth cavity malignancies [195-198] (Figure 6). Furthermore, ethanol-containing mouthwashes do not possess antiplaque properties [199]. Additionally, almost all mouthwashes are inept at pulling out biofilms and ineffective in eliminating the total microbial content of biofilm [200]. Moreover, multiple times rinsing utilizing such high alcohol-containing cleansing agents exposes the oral cavity much more times than those who consume alcoholic beverages and increases the risk of "hyperkerastosic lesions" [199].

Rajendiran et al. reported that the usual active constituents in dental gel or cream and mouth rinse comprise "chlorhexidine, cetylpyridinium chloride, sodium fluoride, stannous fluoride, stannous chloride, zinc oxide, zinc chloride, and two herbs - licorice and curcumin" [201]. Flossing and toothbrushing to conserve oral cleanness and periodontal health are the best endorsed and effectual procedures to prevent plaque formation [201-203]. The areas of the oral cavity that are to a lesser extent reachable to flossing and toothbrushing; chemical plaque control agents (chlorhexidine, cetylpyridinium chloride, amine fluoride/stannous fluoride, stannous chloride, delmopinol hydrochloride, hexetidine, triclosan, phenolic compounds, etc.) containing in dental gel, cream, pastes, and mouthwashes can prevent the evolution of plaque biofilm [201,204-207].

Limitations of the study

The results of this review need to be generalizable, and more clinical and histological studies with large sample sizes across various ethnic populations are required. An increased number of longitudinal studies and systematic reviews need to be carried out to assess the association between alcoholism and periodontal disease. Systemic factors and psychosocial assessment of alcoholism need to be evaluated.

## Conclusions

Alcohol is either directly or indirectly associated with periodontal diseases. It is also one of the significant factors that may cause clinical attachment loss, pocket formation, and dental caries. There is an imbalance of microbes within the oral cavity of alcohol consumers, and the presence of sugar in alcoholic beverages further promotes the build-up of bacteria that aggravate the periodontal disease. Alcohol over-dependency, its toxic effects, undernourishment, and poor immunity may lead to NPD. Human beings who are consuming alcohol for a long time can develop some toxic effects. Alcohol deteriorates an individual's physical, social, psychological, and financial condition. If it is associated with other factors, such as using tobacco, smoking, or excessive sugar intake, it worsens the conditions. An unhealthy oral cavity would contribute to an individual’s reduced quality of life. It is of great importance that the consumption of alcohol is limited and oral health is maintained with flossing, tooth brushing, and, if needed, dental gel, cream, paste, and mouthwash to remove harmful elements within the oral cavity. Public health workers, policymakers, physicians, and dentists need to impart knowledge regarding the health-deteriorating effects of alcohol consumption and need to encourage alcohol consumers to pay regular visits to dentists to keep periodontal diseases at bay.
